# First visual occurrence data for deep-sea cnidarians in the South-western Colombian Caribbean

**DOI:** 10.3897/BDJ.7.e33091

**Published:** 2019-05-07

**Authors:** Luisa F. Dueñas, Cristina Cedeño-Posso, Alejandro Grajales, Santiago Herrera, Estefanía Rodriguez, Juan Armando Sánchez, Jorge Leon, Vladimir Puentes

**Affiliations:** 1 Universidad Nacional de Colombia - Sede Bogotá - Facultad de Ciencias - Departamento de Biología - Carrera 30 No. 45-03 Edificio 421, Bogotá, D.C., Colombia Universidad Nacional de Colombia - Sede Bogotá - Facultad de Ciencias - Departamento de Biología - Carrera 30 No. 45-03 Edificio 421 Bogotá, D.C. Colombia; 2 Anadarko Colombia Company, Calle 113 No. 7-80 piso 11, Bogotá, D.C., Colombia Anadarko Colombia Company, Calle 113 No. 7-80 piso 11 Bogotá, D.C. Colombia; 3 Instituto de Investigaciones Marinas y Costeras- INVEMAR. Calle 25 No. 2-55 Playa Salguero , Santa Marta, Colombia Instituto de Investigaciones Marinas y Costeras- INVEMAR. Calle 25 No. 2-55 Playa Salguero Santa Marta Colombia; 4 Division of Invertebrate Zoology, American Museum of Natural History. Central Park West at 79th Street, New York, NY 10024, United States of America Division of Invertebrate Zoology, American Museum of Natural History. Central Park West at 79th Street New York, NY 10024 United States of America; 5 Department of Biological Sciences, Lehigh University. 111 Research Dr., Bethlehem, PA 18015, United States of America Department of Biological Sciences, Lehigh University. 111 Research Dr. Bethlehem, PA 18015 United States of America; 6 Departamento de Ciencias Biológicas, Universidad de los Andes. Carrera 1 No. 18A-12, Bogotá, D.C, Colombia Departamento de Ciencias Biológicas, Universidad de los Andes. Carrera 1 No. 18A-12 Bogotá, D.C Colombia

**Keywords:** Biodiversity, Benthic, DwC-A, Marine, Remote Operated Vehicle-ROV, Tow Camera, Soft Bottom

## Abstract

**Background:**

Attention to the deep-sea environment has increased dramatically in the last decade due to the rising interest in natural resource exploitation. Although Colombia holds a large submerged territory, knowledge of the seabed and its biodiversity beyond 1,000 m depth is very limited. During 2015–2017, Anadarko Colombia Company (ACC) carried out hydrocarbon exploratory activities in the South-western Colombian Caribbean, at depths between 375 m and 2,565 m.

**New information:**

Capitalising on available data resources from these activities, several cnidarian species were observed in ROV and towed camera surveys. We analysed over nine hours of video and 5,066 still images from these surveys, identifying organisms to the lowest possible taxonomic level. The images and associated data presented here correspond to 108 observations of deep-sea cnidarians, including seven new records for the Colombian Caribbean. Given the paucity of research and funding to explore the deep-sea in Colombia, the present dataset comprises the largest deep-sea Cnidaria imagery inventory to date for the Colombian Caribbean.

## Introduction

Interest in the deep seas has increased dramatically in the last decade, due to the potential presence of natural resources (oil, gas, precious and rare metals, fishery resources, pharmaceuticals, biodiversity etc.). Likewise, there is an increasing concern about the health of this environment, which calls for the acquisition of baseline data (Mengerink et al. 2014). Although Colombia holds a large submerged territory and there has been extensive research on cnidarians from the continental margin and upper slope (Florez and Santodomingo 2010, Santodomingo et al. 2012), knowledge of the seabed below 1,000 m depth is sparse. All previous investigations addressing the characterisation of deep-water ecosystems below 1,000 m have relied on samples taken by box corer or benthic sleds (Polanco F et al. 2017), but no *in-situ* visual confirmations have been provided.

Visual observation of the seabed and its inhabitants is possible using vessels and submersible equipment that can be remote (ROV), autonomous (AUV) or manned. This is a non-invasive *in-situ* procedure that can reach depths and environments that are normally out of human reach (Huvenne et al. 2018). Therefore, visual data (photographs and videos) provide valuable information on the geological characteristics, physical structure and biological components of a benthic habitat (Pawson et al. 2015, Zawada et al. 2007). However, this sampling method is limited only to the identification of large organisms, i.e. megafauna, with no evasive behaviour (Zawada et al. 2007). Furthermore, the quality of the images is highly dependent on the equipment and experience and qualifications of the operator (Solem 2017). In addition, in instances such as the identification some soft-bodied cnidarians like anemones and cerianthids, image data provide limited information allowing the identification at high taxonomic ranks only (i.e. family and genus in some cases). Species-level identification for these particular cases requires a detailed description of internal and microanatomical characters (e.g. Carlgren 1949, England 1987, Häussermann 2004, Rodriguez and López-González 2013), all of which requires specimen sampling and examination.

Video and still photographs are useful for documenting biological and ecological information, species identification, public outreach and scientific publications (Etnoyer et al. 2006). It provides a useful resource that can be made readily available to other scientists, as it was in this case. However, the use of images for organism identification may impose some technical limitations, in particular regarding the quality of the images. High-quality images depend on the camera used during the survey (digital quality, the angle of the setup and zoom), water transparency conditions, the lighting setup (type and angle of the lights) and the file format used to store the image data. Quality also depends on the skill of the engineer who operates the equipment and his/her expertise on the observed organisms. This will determine whether essential morphological features for accurate identification of the organisms (diagnostic characters) could be targeted for close-ups stills. Finally, for some groups of organisms (especially those relying on internal characters for identification, e.g. sea anemones), this type of data provides limited information for reliable identifications (even at high taxonomic levels) and detailed taxonomic descriptions, requiring sampling and morphological observations in order to provide species-level identifications. Nonetheless, such “first-pass” biodiversity surveys provide an invaluable source of information, especially from previously unexplored environments and constitute the foundation for further, more thorough initiatives.

Several types of surveys are essential for the deep-sea hydrocarbon exploratory activities in Colombia. Exploratory drilling activities normally include ROV video surveys before and after drilling. These surveys are critical to evaluate the seabed conditions around the well to minimise potential impacts to sensitive ecosystems (e.g. chemosynthetic communities, deep-sea coral reefs) or archaeological sites. These vehicles are also essential to carry out inspections of the subsea infrastructure to keep the integrity of the well (Gates 2016, Tena 2011). In addition to ROV, detailed characterisation of seabed features and fauna can be obtained from towed camera surveys if needed. Given the paucity of research and funding to explore the deep-sea in Colombia, the present dataset comprises the largest deep-sea Cnidaria imagery inventory to date for the deep sea of Colombian Caribbean.

Cnidarians (jellyfish, corals, sea anemones, amongst others) are one of the most ancient invertebrate groups that keeps a simple body structure with two cell layers and a blind gut (Cairns et al. 2009, Daly et al. 2007). They are found in all aquatic (marine and freshwater) environments, being more diverse in marine habitats. Although simple in body organisation, cnidarians have evolved as specialised carnivores, catching their prey aided by cnidocytes, which are specialised cells with stinging structures, a phylum-defining trait (Technau and Steele 2011). In oceanic waters, cnidarians can be found in nearly all ecosystems from shallow waters to abyssal depths and from polar regions to tropical latitudes. Benthic cnidarians, such as corals colonising both soft and hard substrates, are efficient suspension feeders that provide a tridimensional habitat in the deep-sea (Etnoyer and Morgan 2005), which sustains high numbers of associated invertebrates (Buhl-Mortensen and Mortensen 2005). Cnidarians, particularly anthozoans, are now recognised as major contributors of biogenic environments in the deep-sea (Hourigan et al. 2017). In addition, even the rarest types of pelagic or benthic cnidarians are also found in the deep-sea (Osborn et al. 2007, Miranda et al. 2018). Given the low density of deep-sea fauna in general, numerous surveys are usually required to recognise common vs. rare species. Hydrocarbon drilling activities have provided some of the few opportunities to perform extensive observations in the deep-sea (Gates et al. 2017).

During 2015–2017, Anadarko Colombia Company (ACC), a subsidiary of Anadarko Petroleum Corporation, carried out hydrocarbon exploratory activities in the deep sea South-western Colombian Caribbean. The activities included both ROV and towed camera surveys at depths between 375 m and 2,565 m. Capitalising on the availability of the images obtained from these activities, several organisms from different phyla were spotted and identified to the lowest possible taxonomic status. This is the first of a series of datasets reporting visual confirmations on the occurrences of deep-sea organisms, in this case, 108 cnidarians.

## Sampling methods

### Sampling description

**ROV surveys**

ROV video surveys were performed in a cross pattern. From a central point that could be a transponder (tool for positioning the drilling vessel) or the drilling location, surveys were executed in an 80 metre-long transect with a north trajectory, then an 80 metre-long transect with a south trajectory, an 80 metre-long transect with an east trajectory and finally an 80 metre-long transect with a west trajectory (Fig. 1). Video-transects included soft bottom images, where only one species of a pelagic cnidarian was encountered. Videos were taken before and after drilling for all the exploratory wells, or even during drilling for two of the wells.

**Towed camera surveys**

Survey areas of interest (i.e. Chamana, Chamai, Yaduli, Cana Norte and Cawa) were assessed using 25 towed camera transects. These transects registered seafloor features, during 3-hour surveys, taking still images every 20 seconds. Benthic and pelagic megafauna specimens were recorded in the images.

### Quality control

Videos were analysed twice for the presence of cnidarians by two different experts. This methodology allowed us to ensure all cnidarians were registered. Still images were also analysed twice by the same expert, who did preliminary identification to phylum and class. All cnidarian images were then identified to the lowest possible taxonomic level. When in doubt about the identification, additional experts were contacted and the images were sent to them in jpg format for towed camera photographs and as a snapshot (also in jpg format) for ROV videos.

Given that the acquisition of the images used in this paper was carried out for other purposes and objectives, they do not have the best quality for species identification. Nevertheless, the images were useful, and represent the first visual confirmation of these deep-sea organisms for the Colombian Caribbean.

### Step description

For ROV surveys, we analysed a total of 48 video transects (duration: 9 h 9 min 26 sec), looking for benthic and pelagic cnidarians. We took snapshots of each cnidarian, registered coordinates and depth and identified them to the lowest possible taxonomic level. On the other hand, for towed camera images, we analysed a total of 5,066 photographs, looking for benthic and pelagic cnidarians. For each cnidarian, coordinates and depth were registered and identified to the lowest possible taxonomic level. We cropped the photographs to include only the organism and performed image correction to reduce the bluish colour cast with the *Auto Tone* function in Adobe Photoshop CC 2018 (Fig. 2).

Using photographs and video footage, we highlighted the occurrences for Cnidaria here. Cnidarians were further identified to the lowest possible taxonomic level, based on comparison with literature (Fautin 2016, Molodtsova 2006, Williams 2011, Kramp 1961, Thuesen 2003, Burton and Lundsten 2008), databases (Board, WoRMS Editorial 2017, Fautin 2013) and with the help of taxonomic experts of each group. Taxonomic experts that helped in the identification of the cnidarians include Dennis Opresko (Smithsonian National Museum of Natural History), Mercer Brugler (New York City College of Technology), Tina Molodtsova (P.P. Shirshov Institute of Oceanology), Frederic Sinniger (University of the Ryukyus) and Steven Haddock (Monterey Bay Aquarium Research Institute). The taxonomy presented here is in accordance with the World Register of Marine Species – WORMS (http://www.marinespecies.org/).

## Geographic coverage

### Description

**Figure 3.**
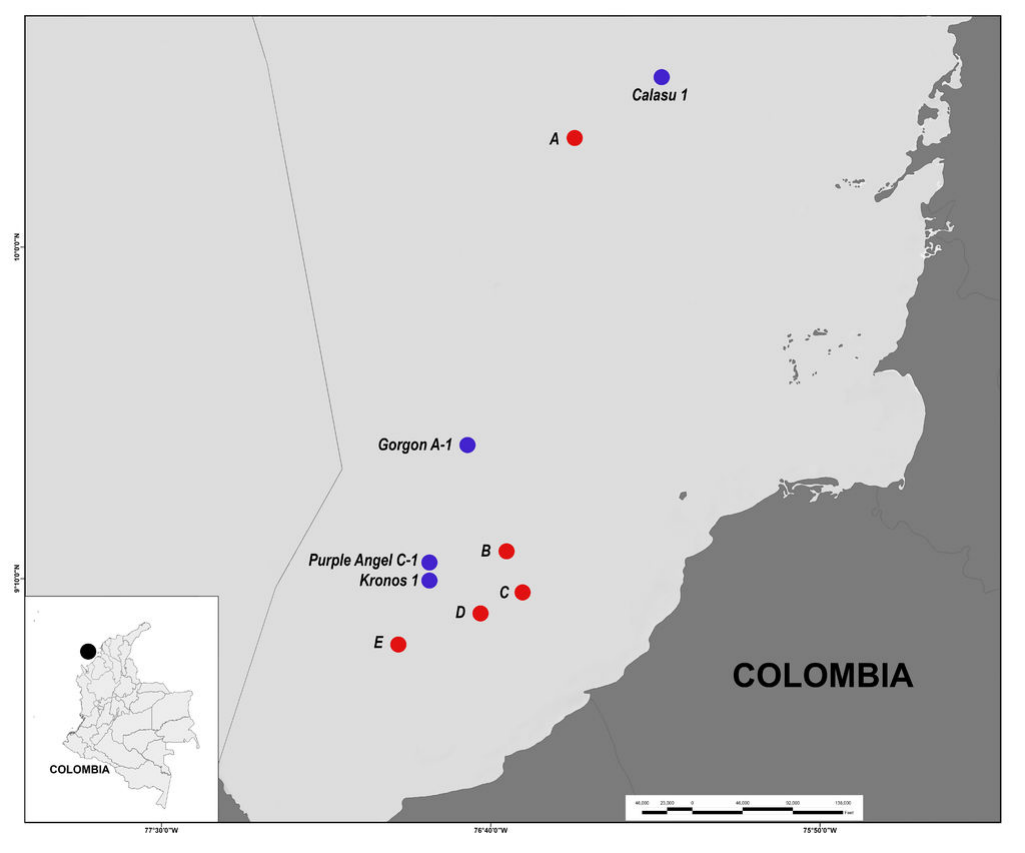
Geographical location of the sites surveyed at the South-western Colombian Caribbean. Blue circles depict the wells and red circles correspond to other surveyed areas.

ACC’s deep sea hydrocarbon exploratory activities in the South-western Colombian Caribbean included four exploratory wells (Calasu 1, Kronos 1, Gorgon A-1 and Purple Angel C-1) and five other survey areas of interest (A-E), at depths between 375 m and 2,565 m (Fig. 3). Wells and areas of interest are approximately 54 to 74 km offshore from the nearest point in the Caribbean coast of Colombia.

### Coordinates

9.27 and 10.369 Latitude; -76.481 and -76.932 Longitude.

## Taxonomic coverage

### Description

The data presented here correspond to 108 occurrences of deep-sea cnidarians from the South-western Colombian Caribbean, spotted over soft bottoms at depths between 375 to 2,565 m. The dataset contains the original data of depth, geographical coordinates, date and hour of the event, for each image that registered a cnidarian. Additional information includes the methodology used to obtain the images (see Methods section), taxonomic identification to the lowest possible taxonomic level, the name of the expert who identified the organism and the number of individuals of the species per image. The dataset also contains an extension with links to the images supporting the occurrences.

Seven occurrences were obtained through the ROV surveys, all corresponding to the pelagic jellyfish Poralia cf. rufescens Vanhöffen, 1902. Tow camera occurrences (n = 101) registered members of the classes Scyphozoa (n = 1), Hydrozoa (n = 13) and Anthozoa (n = 87). Only two orders of jellyfish, Trachymedusae (*Crossota millsae* Thuesen, 2003 and *Voragonema pedunculata* (Bigelow, 1913)) and Narcomedusae represent Hydrozoa, while members of seven orders represented Anthozoa. Within Anthozoa, organisms from the three subclasses (Hexacorallia, Octocorallia and Ceriantharia) were spotted. Hexacorallia was represented by anemones (order Actiniaria), the zoanthid Epizoanthus cf. stellaris Hertwig, 1888 (order Zoantharia), the black coral Bathypathes cf. patula Brook, 1889 (order Antipatharia) and cerianthids (order Ceriantharia). Octocorals were represented by the orders Alcyonacea and Pennatulacea, the latter with seven organisms belonging to the genus *Umbellula* Gray, 1870. Finally, corallimorphs (order Corallimorpharia) were represented by the genus *Corallimorphus* Moseley, 1877 (Table 1).

Based on the 101 occurrences from towed camera surveys, we registered eight new reports of cnidarians for the Colombian Caribbean. The new records for the area comprised two sea anemones, one zoanthid, one corallimorpharian, one octocoral, one black coral (Fig. 4) and two jellyfish species (Fig. 5).

The genus *Corallimorphus* has a total of six valid species (Fautin 2016), present at all latitudes in deep waters (Fautin 2011), with the exception of *C. profundus*, which has been identified from shallow environments in Antarctica (Riemann-Zürneck and Iken 2003). Two species of the genus, *C. ingens* and *C. rigidus*, have been reported in the Atlantic Ocean (i.e. Den Hartog et al. 1993, Ocaña and Den Hartog 2002). Detailed records of members of the family Corallimorphidae are available for the greater Caribbean region and the Gulf of Mexico (Fautin and Daly 2009, González-Muñoz et al. 2013, González-Muñoz et al. 2016), but are limited to shallow water species. A study of the deep-sea communities off the east coast of Florida reports a record of *Corallimorphus* sp. based on still photographic data (Messing et al. 2012). With the available information, the authors were not able to classify the specimens beyond the genus level.

The genus *Phelliactis* (fam. Hormathiidae) is composed of 20 valid species distributed worldwide (Fautin 2016). In the Caribbean, records for species of the genus exist from hydrothermal vent communities in the Cayman Islands (Plouviez et al. 2015) and deep-sea benthos of Venezuela (Briggs et al. 1996). This last study reports the presence of *P. michaelsarsi*, effectively expanding the distribution range for the species, currently limited to the East and Mid Atlantic Ocean (Molodtsova et al. 2008). Unfortunately, this report does not provide enough information regarding the identification methodology employed by the authors in order to determine this species identity (i.e. geographical proximity, detailed morphological description), thus we cannot evaluate the validity of this record.

Anemones, belonging to the genus *Adamsia*, are known for holding symbiotic relationships with hermit crabs, most commonly with the species *Sympagurus pictus* (Daly et al. 2004), but reports also exist for the species *Parapagurus pilosimanus* and the gastropods of the genus *Oocorys* (Ammons and Daly 2008). There are a total of 6 valid species (Fautin 2016) with *A. obvolva* being the only species reported in the region, specifically in several localities from the Gulf of Mexico (Daly et al. 2004, Ammons and Daly 2008). The bathymetric range of the specimens reported here (Table 1) is larger than the range reported for *A. obvolva* (300 to 800 m). The records, presented here, would correspond to an expansion of the species’ geographic and bathymetric ranges; however, it was not possible to confirm its identity. Even the examination of diagnostic external anatomical characters, such as the presence of cinclides (small insertions occurring in the column of the anemone), requires detailed observation under the stereomicroscope.

Other hexacorallians, described as new reports for the Colombian Caribbean, include Bathypathes cf. patula and Epizoanthis cf. stellaris. *Bathypathes patula* is also a widespread, cosmopolitan species that has been described from the Pacific, the Atlantic, the Indian Ocean, the Gulf of México and Puerto Rico (Molodtsova 2006, Horowitz et al. 2018). It belongs to the Antipatharian genus *Bathypathes* Brook, 1889 (Anthozoa: Hexacorallia) which currently holds 19 species (Molodtsova and Opresko 2019). *Bathypathes patula* is characterised by a wide vertical distribution that ranges from 100 m to 5500 m in depth (Molodtsova 2006). On the other hand, the zoanthid genus *Epizoanthus* Gray, 1867 (Anthozoa: Hexacorallia) is a rich genus with 105 species and a global distribution (Reimer and Sinniger 2019, Kise and Reimer 2016). Particularly, *Epizoanthis stellaris* Hertwig, 1888 has been reported to grow over the siliceous spicules of glass sponges (Beaulieu 2001), as also seen in this study.

The pennatulacean genus *Umbellula* comprises 13 species (Cordeiro et al. 2019) and belongs to the monogeneric family Umbellulidae (Anthozoa: Octocorallia). This genus has a widespread to nearly worldwide distribution and is the deep-sea pennatulacean genus with the widest vertical distribution reaching depths of 6,000 m (Williams 2011). The most common species is *U. lindahi* that has a cosmopolitan distribution in all of the world's oceans (Tyler et al. 1995).

Finally, the two hydromedusae from the Rhopalonematidae family, *Crossota millsae* and *Voragonema pedunculata* (previously know as *Benthocodon pedunculata*), are bathypelagic and often found in groups drifting above the bottom; this is the reason why they are also described as benthopelagic. These two species have been reported in the Western Atlantic (the Bahamas and Dry Tortugas) (Larson et al. 1991, Larson et al. 1992) and the Gulf of Mexico (Valentine and Benfield 2013).

## Temporal coverage

**Data range:** 2015-2-24 – 2017-4-26.

## Usage rights

### Use license

Other

### IP rights notes

Creative Commons Attribution Non Commercial (CC-BY-NC) 4.0 License

## Data resources

### Data package title

First visual occurrence data for deep-sea cnidarians in the South-western Colombian Caribbean

### Resource link

https://doi.org/10.15472/o8xonn

### Alternative identifiers

https://ipt.biodiversidad.co/sibm/resource?r=anadarko_colombia_002

### Number of data sets

1

### Data set 1.

#### Data set name

First visual occurrence data for deep-sea cnidarians in the South-western Colombian Caribbean

#### Data format

Darwin Core Archive (DwC-A)

#### Number of columns

60

#### Character set

UTF-8

#### Description

The data presented here corresponds to occurrences of deep-sea cnidarians from the South-western Colombian Caribbean, spotted over soft bottoms at depths between 375 to 2,565 m.

**Data set 1.** 

Column label	Column description
occurrenceID	An identifier for the Occurrence
basisOfRecord	The specific nature of the data record
institutionCode	The name in use by the institution having custody of the information referred to in the record
collectionCode	The name, acronym, coden or initialism identifying the collection or dataset from which the record was derived
catalogNumber	An identifier for the record within the dataset
type	The nature or genre of the resource
language	Thelanguage of the resource
rightsHolder	The organisation owning or managing rights over the resource
accessRights	Information about who can access the resource or an indication of its security status
institutionID	An identifier for the institution having custody of the information referred to in the record.
ownerInstitutionCode	The name in use by the institution having ownership of the information referred to in the record
recordedBy	People responsible for recording the original Occurrence
individualCount	The number of individuals represented present at the time of the Occurrence
lifeStage	The age class or life stage of the biological individual(s) at the time the Occurrence was recorded
occurrenceStatus	A statement about the presence or absence of a Taxon at a Location
preparations	A list of preparations and preservation methods for a specimen
samplingProtocol	Protocol used during an Event
samplingEffort	The amount of effort expended during an Event
eventDate	The date during which an Event occurred
eventTime	The time during which an Event occurred
year	The four-digit year in which the Event occurred
month	The ordinal month in which the Event occurred
day	The integer day of the month on which the Event occurred
verbatimEventDate	The verbatim original representation of the date and time information for an Event
habitat	A category or description of the habitat in which the Event occurred
higherGeography	Geographic location less specific than the information captured in the locality term
continent	The name of the continent in which the Location occurs
waterBody	The name of the water body in which the Location occurs
locationID	An identifier for the set of location information
country	The name of the country in which the Location occurs
countryCode	The standard code for the country in which the Location occurs
locality	The specific description of the place
verbatimDepth	The original description of the depth below the local surface
minimumDepthInMetres	The lesser depth of a range of depth below the local surface, in metres
maximumDepthInMetres	The greater depth of a range of depth below the local surface, in metres
verbatimLatitude	The verbatim original latitude of the Location
verbatimLongitude	The verbatim original longitude of the Location
verbatimCoordinateSystem	The spatial coordinate system for the verbatimLatitude and verbatimLongitude or the verbatimCoordinates of the Location
decimalLatitude	The geographic latitude of the geographic centre of a Location
decimalLongitude	The geographic longitude of the geographic centre of a Location
geodeticDatum	The ellipsoid, geodetic datum or spatial reference system (SRS) upon which the geographic coordinates given in decimalLatitude and decimalLongitude as based
georeferencedBy	The organisation who determined the georeference for the Location
identifiedBy	A list of names of people who assigned the Taxon to the subject
dateIdentified	The date on which the subject was identified as representing the Taxon
identificationQualifier	A brief phrase or a standard term to express the determiner's doubts about the Identification
scientificNameID	An identifier for the nomenclatural details of a scientific name
nameAccordingToID	An identifier for the source in which the specific taxon concept circumscription is defined or implied
acceptedNameUsage	The full name, with authorship and date information if known, of the currently valid (zoological) or accepted (botanical) taxon
nameAccordingTo	The reference to the source in which the specific taxon concept circumscription is defined or implied - traditionally signified by the Latin "sensu" or "sec."
scientificName	The full scientific name, with authorship and date information if known
kingdom	The full scientific name of the kingdom in which the taxon is classified
phylum	The full scientific name of the phylum or division in which the taxon is classified
class	The full scientific name of the class in which the taxon is classified
order	The full scientific name of the order in which the taxon is classified
family	The full scientific name of the family in which the taxon is classified
genus	The full scientific name of the genus in which the taxon is classified
specificEpithet	The name of the first or species epithet of the scientificName
taxonRank	The taxonomic rank of the most specific name in the scientificName
scientificNameAuthorship	The authorship information for the scientificName
taxonomicStatus	The status of the use of the scientificName as a label for a taxon

## Figures and Tables

**Figure 1a.**
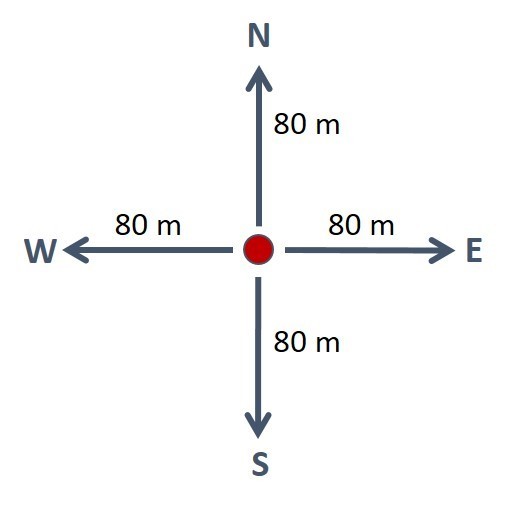
Schematic representation of the ROV transects is cross-fashion. Red circle denotes the location of the well or transponder.

**Figure 1b.**
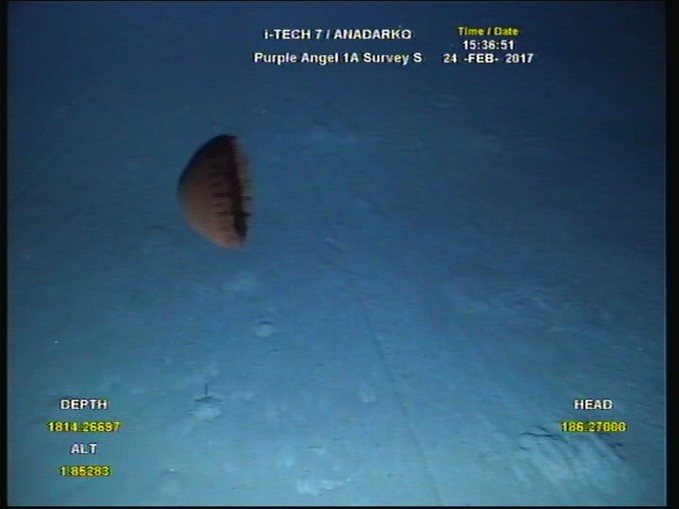
Example of a snapshot taken from the ROV video, depicting a specimen of *Poralia rufescens*.

**Figure 2a.**
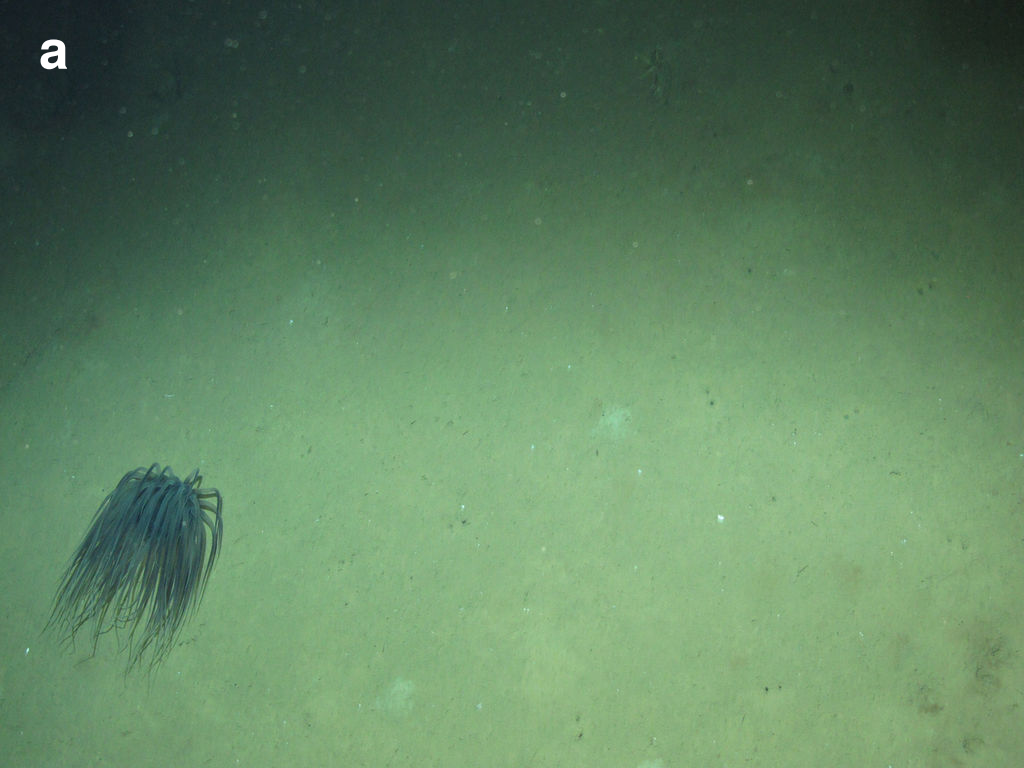
Original photograph of a cerianthid (subclass Ceriantharia) obtained from the tow camera.

**Figure 2b.**
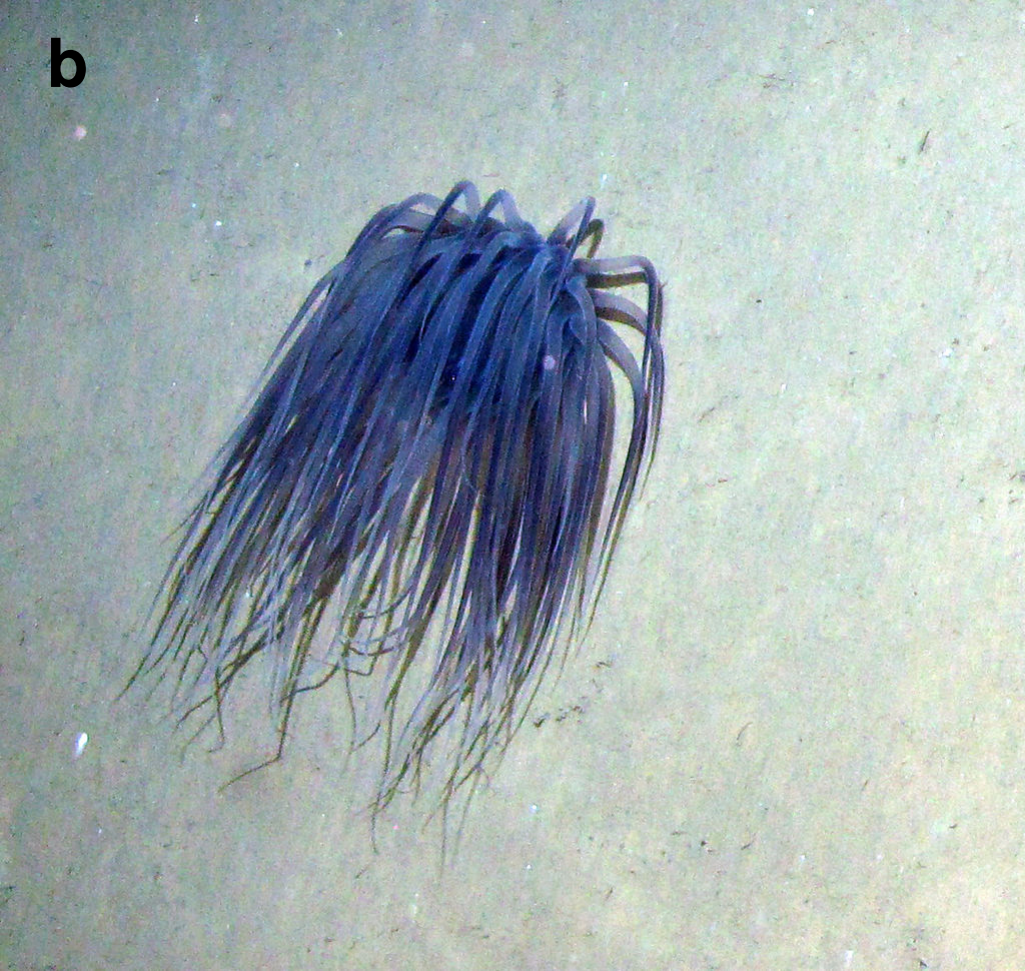
Cropped photograph that includes only the organism and colour cast corrected using the *Auto Tone* function.

**Figure 4a.**
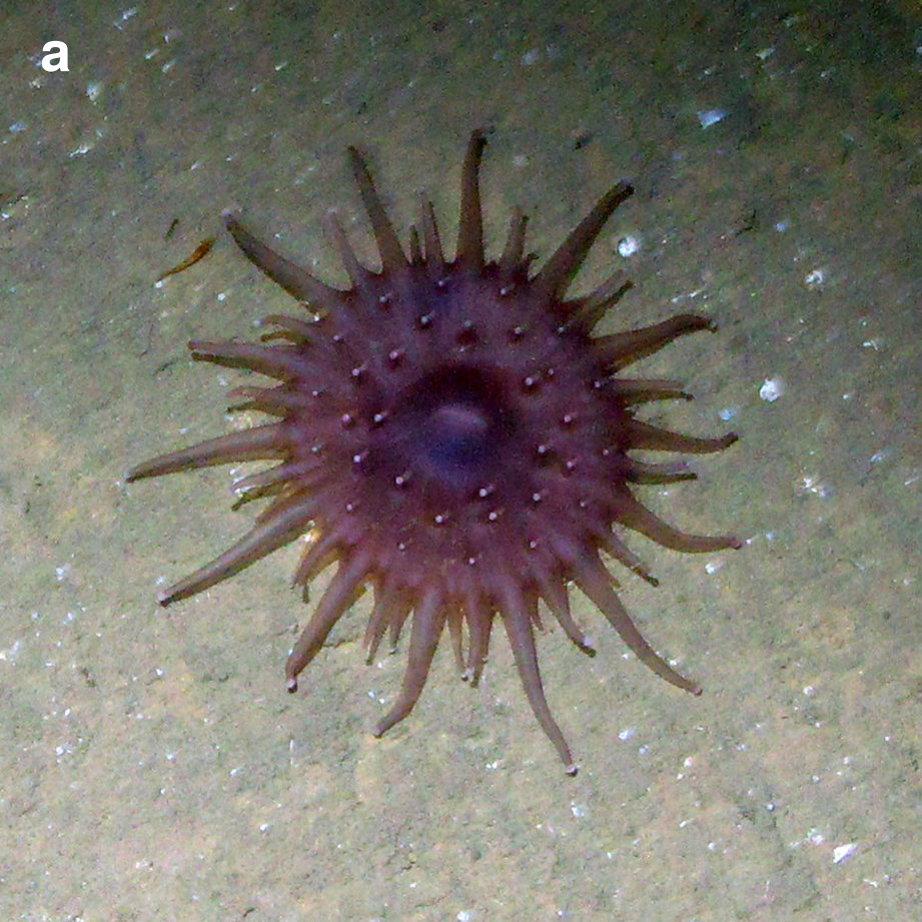
*Corallimorphus* sp.

**Figure 4b.**
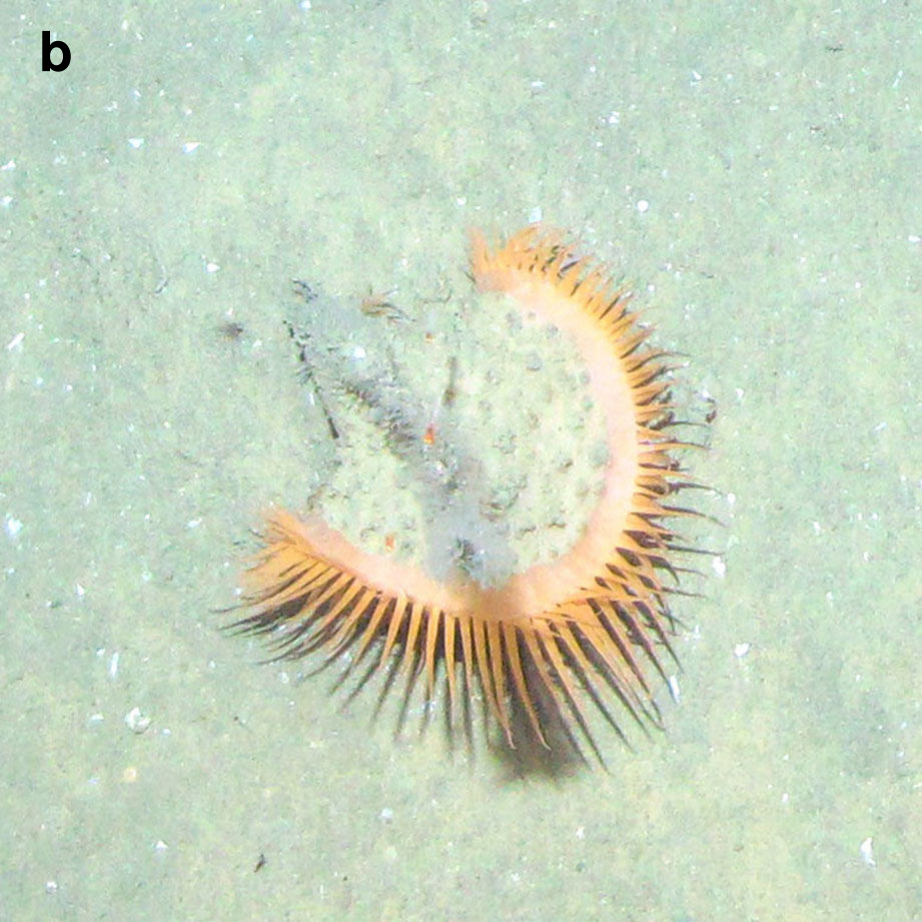
*Phelliactis* sp.

**Figure 4c.**
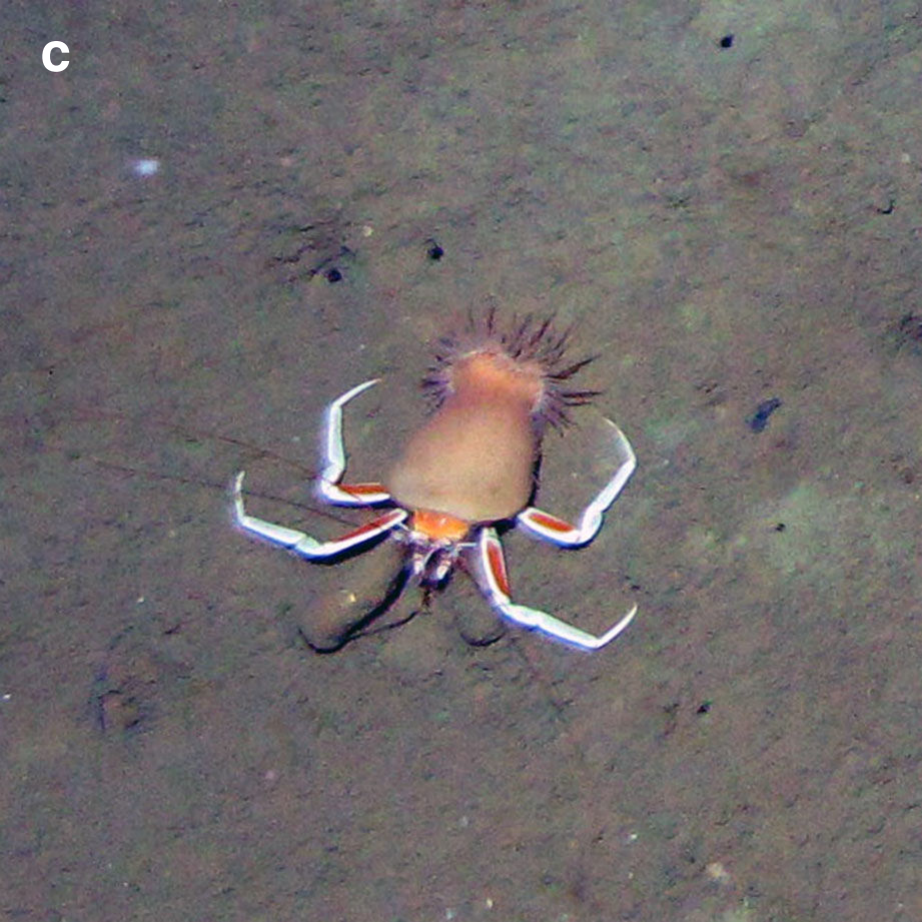
*Adamsia* sp. on a hermit crab (*Sympagurus pictus*)

**Figure 4d.**
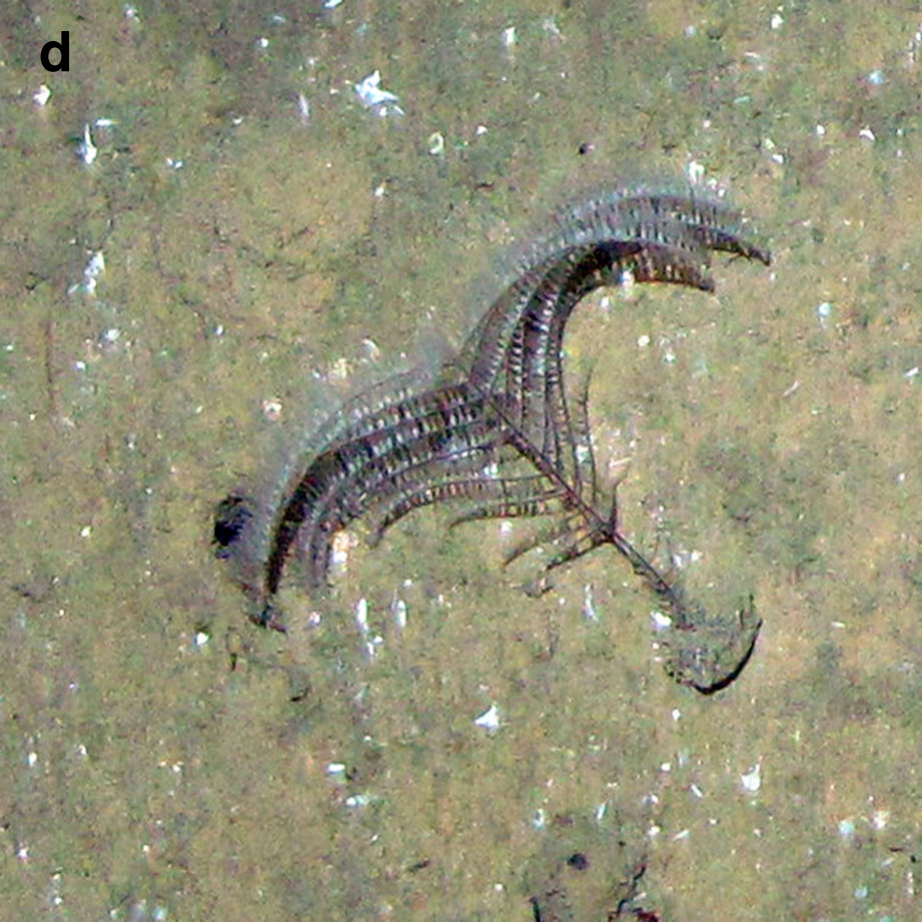
Bathypathes cf. patula

**Figure 4e.**
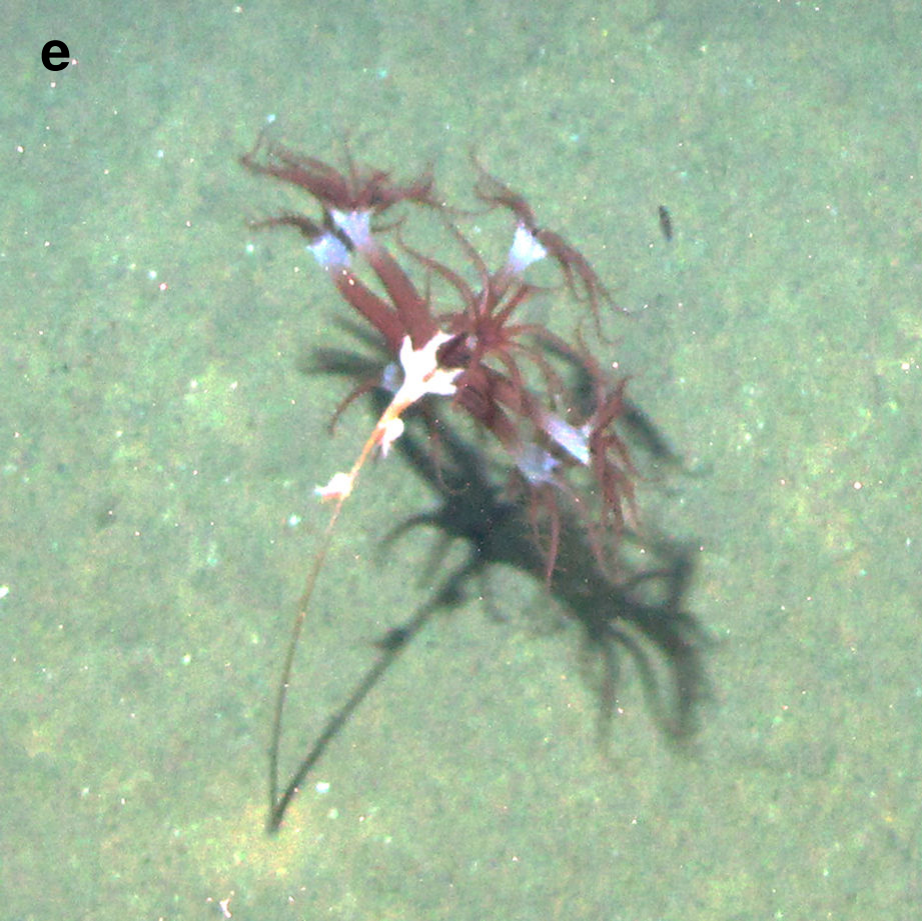
*Umbellula* sp.

**Figure 4f.**
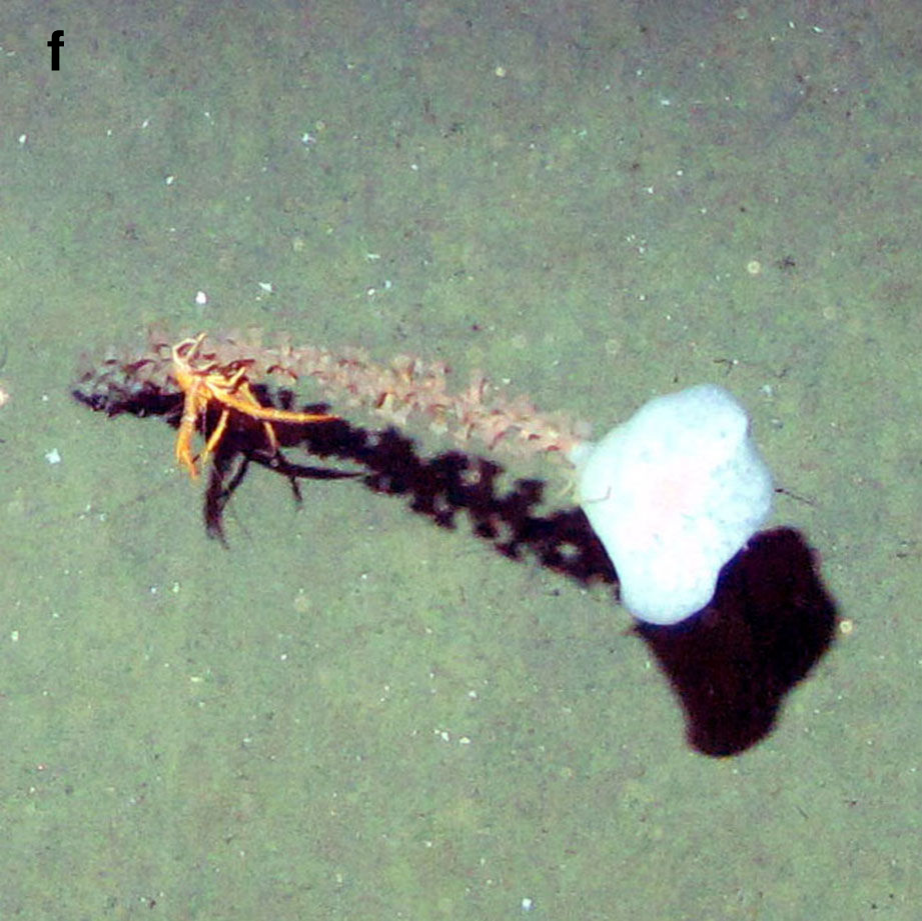
Epizoanthus cf. stellaris growing over a glass sponge (*Hyalonema* sp.) stalk

**Figure 5a.**
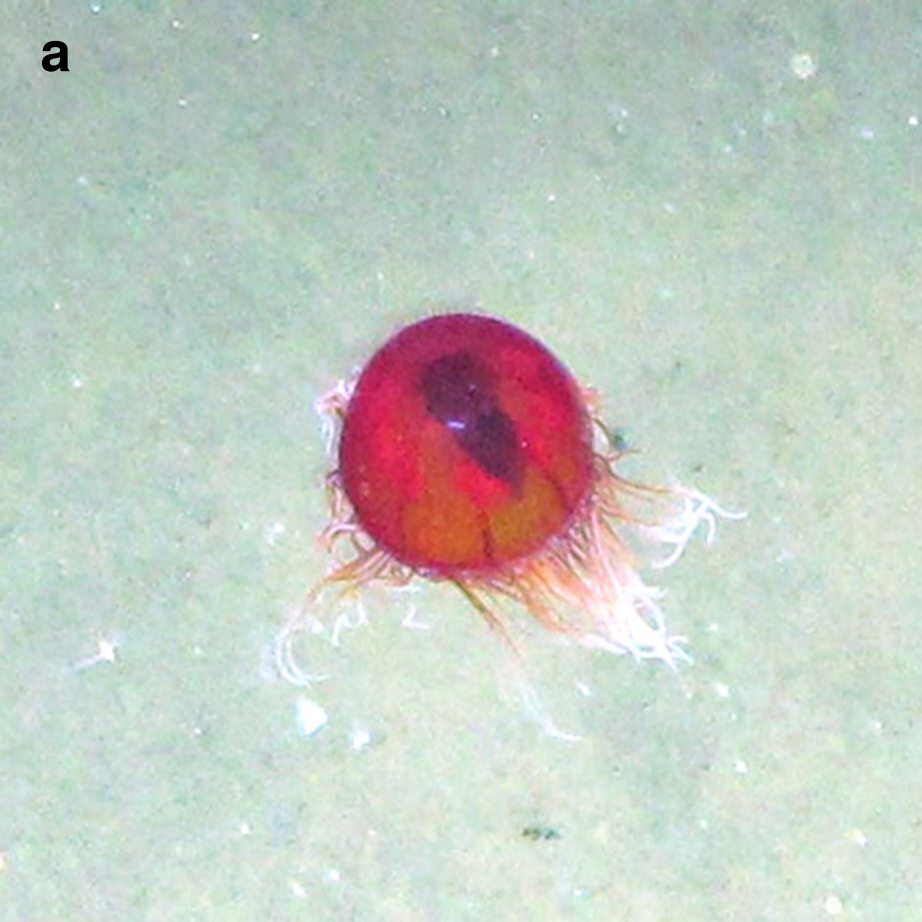
*Crossota millsae*

**Figure 5b.**
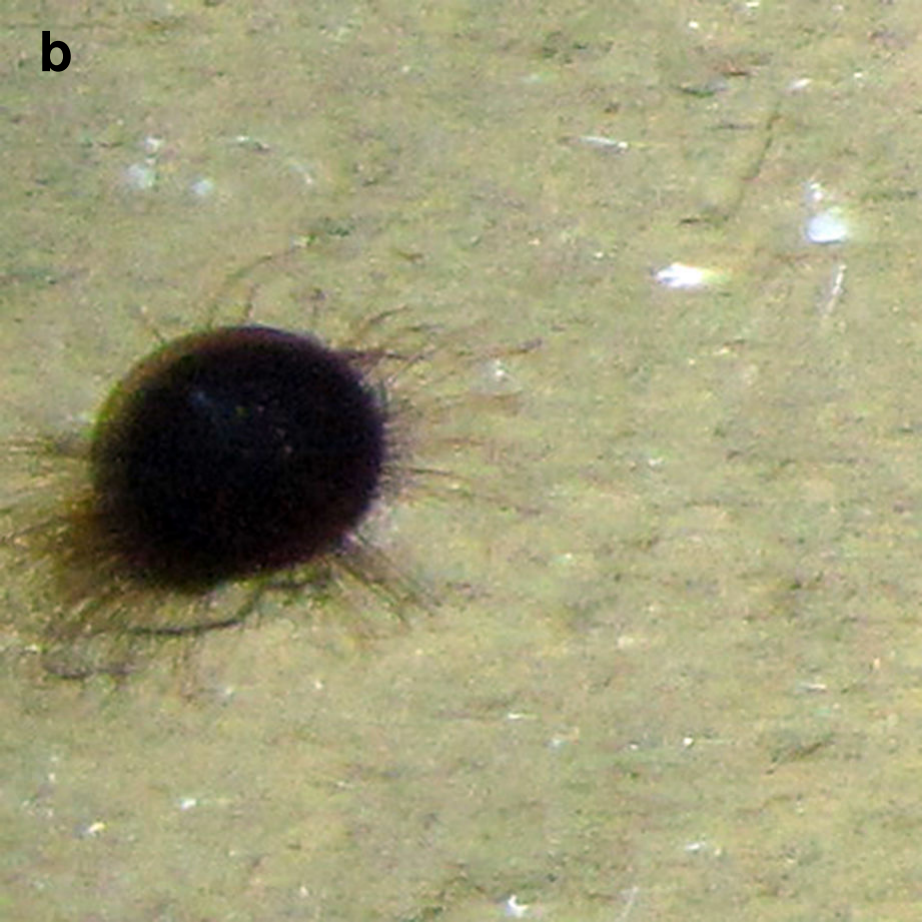
*Voragonema pedunculata*

**Table 1.** Cnidarians found in the deep waters of the South-western Colombian Caribbean. * New reports for the Colombian Caribbean.

**Species**	**Sampling Protocol**	**Depth range (m)**
Poralia cf. rufescens Vanhöffen, 1902	ROV	1,565–1,818
Hydrozoa	Towed Camera	1,839–2,367
Semaeostomeae	Towed Camera	2,289
*Crossota millsae* Thuesen, 2003*	Towed Camera	1,165–1,189
*Voragonema pedunculata* (Bigelow, 1913)*	Towed Camera	2,340–2,562
Narcomedusae	Towed Camera	1,717
Actiniaria	Towed Camera	539–2,523
Ceriantharia	Towed Camera	428–2,058
*Corallimorphus* Moseley, 1877*	Towed Camera	2,259–2,526
Hormathiidae Carlgren, 1932	Towed Camera	375–2,524
Kadosactinidae Riemann-Zürneck, 1991	Towed Camera	2,352–2,430
Bathypathes cf. patula Brook, 1889*	Towed Camera	2,359–2,564
Virgulariidae Verrill, 1868	Towed Camera	654–2,565
*Trichogorgia lyra* Bayer & Muzik, 1976	Towed Camera	577–650
*Umbellula* Gray, 1870*	Towed Camera	1,633–2,525
Chrysogorgiidae Verrill, 1883	Towed Camera	490
Epizoanthus cf. stellaris Hertwig, 1888*	Towed Camera	502–1,196
*Adamsia* Forbes, 1840*	Towed Camera	486–2,563
*Phelliactis* Simon, 1842*	Towed Camera	2,561
